# A landscape assessment of CTSA evaluators and their work in the CTSA consortium, 2021 survey findings

**DOI:** 10.1017/cts.2024.526

**Published:** 2024-04-25

**Authors:** Verónica Hoyo, Eric Nehl, Ann Dozier, Jillian Harvey, Cathleen Kane, Anna Perry, Elias Samuels, Susanne Schmidt, Joe Hunt

**Affiliations:** 1 Northwestern University Clinical and Translational Sciences Institute (NUCATS), Northwestern University, Chicago, IL, USA; 2 Georgia CTSA, Emory University, Atlanta, GA, USA; 3 University of Rochester Clinical and Translational Science Institute, Rochester, NY, USA; 4 MUSC South Carolina Clinical and Translational Science Institute, Charleston, SC, USA; 5 NYU Langone Health Clinical and Translational Science Institute, New York, NY, USA; 6 Wake Forest Clinical and Translational Science Institute, Winston-Salem, NC, USA; 7 Michigan Institute for Clinical and Health Research (MICHR), University of Michigan, Ann Arbor, MI, USA; 8 UT Health San Antonio, Institute for Integration of Medicine and Science, San Antonio, TX, USA; 9 Indiana Clinical and Translational Science Institute, Indianapolis, IN, USA

**Keywords:** Continuous improvement, CTSA, evaluation, NCATS, survey, translational science

## Abstract

This article presents a landscape assessment of the findings from the 2021 Clinical and Translational Science Award (CTSA) Evaluators Survey. This survey was the most recent iteration of a well established, national, peer-led systematic snapshot of the CTSA evaluators, their skillsets, listed evaluation resources, preferred methods, and identified best practices. Three questions guided our study: who are the CTSA evaluators, what competencies do they share and how is their work used within hubs. We describe our survey process (logistics of development, deployment, and differences in historical context with prior instruments); and present its main findings. We provide specific recommendations for evaluation practice in two main categories (National vs Group-level) including, among others, the need for a national, strategic plan for evaluation as well as enhanced mentoring and training of the next generation of evaluators. Although based on the challenges and opportunities currently within the CTSA Consortium, takeaways from this study constitute important lessons with potential for application in other large evaluation consortia. To our knowledge, this is the first time 2021 survey findings are disseminated widely, to increase transparency of the CTSA evaluators' work and to motivate conversations within hub and beyond, as to how best to leverage existent evaluative capacity.

## Introduction

Since its foundation, the Clinical and Translational Science Award (CTSA) Program has been dedicated to a culture of evaluation and continuous improvement [1]. Data-driven, science-based approaches were precisely the link that integrated CTSA hubs under the leadership of the National Institutes of Health (NIH) to the National Center for Advancing Translational Science (NCATS) (whose founding mission is to catalyze the development of health interventions and to bring more, and faster, treatments to patients) [2–3.]

Beginning in 2009, a survey of CTSA evaluators has provided a snapshot of the evaluators and their skillsets, identified evaluation best practices, listed evaluation resources and methods used, and the impact of evaluation across the CTSA hubs [4–5]. Previous results have provided actionable insights on changes in evaluation services, and availability of resources. Although it is necessary to recognize the importance of past achievements in translational research and science, it is equally essential to identify the current efforts and future themes that will shape a new CTSA evaluation agenda.

This article offers a *landscape assessment* of the findings of the 2021 national CTSA Evaluators survey and presents recommendations for evaluation practice considering challenges and opportunities for Evaluators at CTSA Consortium [6]. Special circumstances surrounding the 2021 survey merit a closer look. First, it took place in the midst of the COVID-19 pandemic which undeniably impacted workflows and processes across the hubs but, simultaneously, it offered the opportunity for observing the evaluators’ abilities to adapt and pivot [7]. Second, a new Funding Opportunity Announcement (FOA) for the CTSA Consortium was released during the survey’s field time. Compared to prior FOAs, the new FOA clearly stipulates specific evaluation tasks. Namely, each hub must have a continuous quality improvement program and formal dissemination and implementation activities with related evaluation implications, and maintain oversight and review of ongoing translational science pilot grants [8]. The most recent FOA also emphasizes Clinical and Translational Science (CTS), which necessitates the design and administration of new evaluation plans. These two significant events provide background context to the circumstances at the time of the survey and, although it is impossible to determine how much they directly impacted our peers’ responses, we can confidently say that both were in their consideration.

This short communication is organized into two parts: the first describes the survey itself (logistics of development, deployment, and its differences in historical context with prior instruments) with the second section presenting main findings. As evaluators, we see great value in continuing with this now iterative, well-established survey and we look forward to advancing in the themes identified through the contributions of our fellow CTSA evaluators.

## The 2021 CTSA evaluators survey: development, deployment, and historical survey context

The 2021 CTSA Evaluators Survey was initially designed to provide a landscape assessment of the evaluators themselves and of the most common evaluation methods/frameworks, resources, practices, and data collection processes used throughout the Consortium. As part of its internal operations, the CTSA Consortium has established a series of Program Groups tasked with executing the recommendations of the NCATS Advisory Council Working Group and/or the IOM Report on the CTSA Program. One such group is the Evaluators Group which provides an arena for cross-hub collaborations, sharing of best practices, and topically oriented research groups. In this light, the Program Evaluators Group established an Evaluator Survey Working Group, composed of volunteers from thirteen CTSA hubs, to develop the 2021 questionnaire [9].

The survey questionnaire was based on previous iterations of this peer-led, independent data collection process. The “traditional core” of the survey remained of continued interest to the CTSA evaluator community. Additional questions were added as a result of a collaborative effort that identified new areas of interest. The four primary sections of the survey included: (1) Evaluation Profile: CTSA Hub and Evaluation Team Characteristics including hub age and size, number of team members, evaluation team director’s education level, and, evaluation FTE commitment, (2) Evaluation Resources and Scope, namely, team’s expertise and willingness to provide mentoring, evaluation team’s contribution to CTSA hub performance, progress report, data, and resource allocation decisions, (3) Evaluation Tools and Techniques, i.e., tracking and strategic planning, evaluation methods and tools, and (4) Evaluation Best Practices, Challenges and Special Topics including evaluation challenges for the CTSA hub and achievements for the CTSA hub. Additional survey items, for a total of 44 questions, were included to provide context to questions regarding hubs’ COVID-19 response and its associated effects on their evaluation activities; impact evaluation practices and the development and dissemination of evaluation products. From a survey design perspective, best practices were followed, and every effort was made to reduce survey burden and non-response as well as to maximize data quality [10].

The 2021 National Evaluators Survey was (like its predecessor surveys conducted in 2009, 2010, 2013, and 2018), a census survey of all evaluation programs currently funded within the CTSA Consortium. The online self-administered questionnaire was distributed to the list of contact evaluators on the NCATS CTSA Program Evaluators Group in July 2021. Field Time for the survey was six (6) weeks and five follow-up reminders were used. Out-of-date email addresses were identified and replaced with valid contacts. Direct engagement from members of the Evaluator Survey Working Group with non-responsive hubs was also employed to encourage survey completion. These efforts resulted in a high cooperation rate (96%, 59 hubs completed the survey). To protect anonymity, the fifty-nine CTSA hubs that submitted survey responses were classified according to two main criteria: by age of hub and size of the CTSA hub. Hub age was a function of the initial date of funding from the National Institutes of Health (NIH). Six hub age groups were created from 2006 to 2018 and there were three hub sizes corresponding to the total amount of NCATS-awarded hub funding (small, medium, and large). Whenever pertinent (for example, staffing levels, evaluation tools, etc.), descriptive statistics included comparisons between 2018 and 2021 findings. In all instances, data trends were consistent, see supplemental material for additional details.

## Evaluation profile: CTSA hub and evaluation team characteristics

### CTSA hub characteristics

Of the 59 responding CTSA Hubs, 18 (31%) were classified as large, 15 (25%) as medium, and 26 (44%) as small. By age, 10 (17%) were established in 2006, 11 (19%) in 2007, 11 (19%) in 2008, 12 (20%) in 2009-2010, 8 (14%) in 2011–2012, and 7 (12%) from 2013–2019.

The survey asked respondents to provide a description of their hub composition by indicating the number of different organizations with which they were partnered. Most CTSAs stated having four or five different types of partner organizations: academic medical centers, 50 (85%); universities, 50 (85%); medical schools, 48 (81%), hospital systems, 46 (78%), major medical group practices, 24 (41%), and, others including VA centers, private research centers, community organizations, cooperative extensions, public health organizations, and blood centers, 16 (27%).

### Evaluation team characteristics

The evaluators at the CTSA are a highly educated workforce. Although it is well established that the pathways to evaluation work are varied, and that evaluation theory and practice are distinct, a highly educated workforce is an initial advantage [11]. For the evaluation director, 54 hubs provided data. Of these, 45 83% of CTSA hubs reported having a doctoral degree (PhD or MD), 7 (13%) reported a masters only, and 2 (4%) reported other. In terms of the duration of the evaluation director in their position, results indicate that most (68%) assumed the role after the hub’s establishment. For these evaluation directors post-hub establishment, the most frequently reported length in position was 1–3 years (35% of hubs; finding was fairly consistent across all size categories).

**Figure 1.**
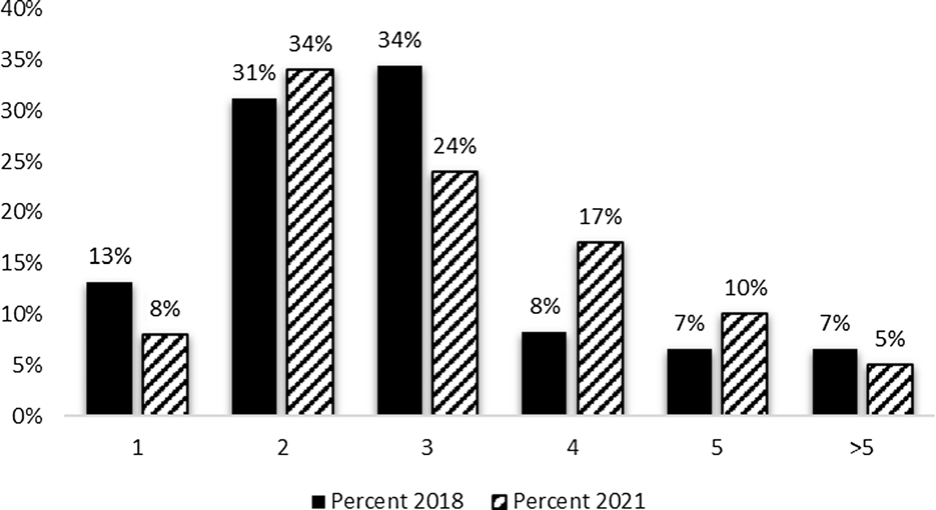
Evaluation team members 2018 and 2021. The mean number of full-time equivalent (FTE) among survey respondents was 1.57 FTE.

The survey sought information regarding both the number of employees on the evaluation team and the net full-time equivalent (FTE)s devoted to conducting hub evaluation efforts. As can be seen in Figure 1, CTSA evaluator teams typically consist of one to three team members. The most frequently reported number of staff on the hub evaluation team was two (34%); followed by three (24%) and four (17%). Although there was a five percent decline in the percentage of hubs with one evaluator and a ten percent reduction in three-person teams, the 2021 results are consistent with 2018 findings.

Evaluators who are based within academic medical centers often have time dedicated to multiple projects, resulting in their efforts being divided (e.g., teaching, other grants and contracts, service). In addition to the number engaged in evaluation efforts at the hub, respondents reported the total full-time equivalent effort for evaluation. Current results indicate that over 60 percent of the hubs reported FTEs between 0.5 and 2.0, indicating that those evaluation team members seen in Figure 1 are likely to have their time split across other efforts. FTE allocations devoted to hub evaluation efforts are similar to those reported in 2018 (data not shown).

The CTSA evaluators as a group are very collaborative in nature: 88% of survey respondents stated having frequently collaborated —internally and externally— in the past 12 months, which is consistent with other CTSA evaluator survey results (100% of the 2006 cohort, 82% of the 2008 cohort). Over 50% of the collaborations included other CTSA hubs. This percentage was consistent across all sizes and age categories. This was an important number given the impact of the COVID-19 pandemic and the shutdown of activities and another testimonial published concerning the adaptive capacity of the group [12–19].

### Evaluation resources and scope

An important feature of the evaluator surveys has been the tracking of evaluation expertise available for CTSA hubs and the employment of that expertise to support hub efforts. As can be seen from Table 1, (1) quantitative analysis, (2) database development and data extraction, and (4 categories tied in third place) data visualization, evaluation designs, qualitative analysis, and survey methods were the top reported evaluation areas of expertise in 2021. These expertise areas are largely consistent with the use of evaluation expertise within the hubs (second column on the right).

**Table 1.** Top 10 reported Clinical and Translational Science Award evaluators’ areas of expertise and their use

Area of expertise	% Having expertise	% Used expertise
Quantitative Analysis	98	86
Database Development and Data Extraction	95	88
Data Visualization	93	88
Evaluation Designs	93	76
Qualitative Analysis	93	80
Survey Methods	93	86
Bibliometric Analysis	91	78
Dissemination and Implementation	91	81
Strategic Analysis	91	83
Impact Analysis	88	68
Mixed Methods	88	75

**Figure 2.**
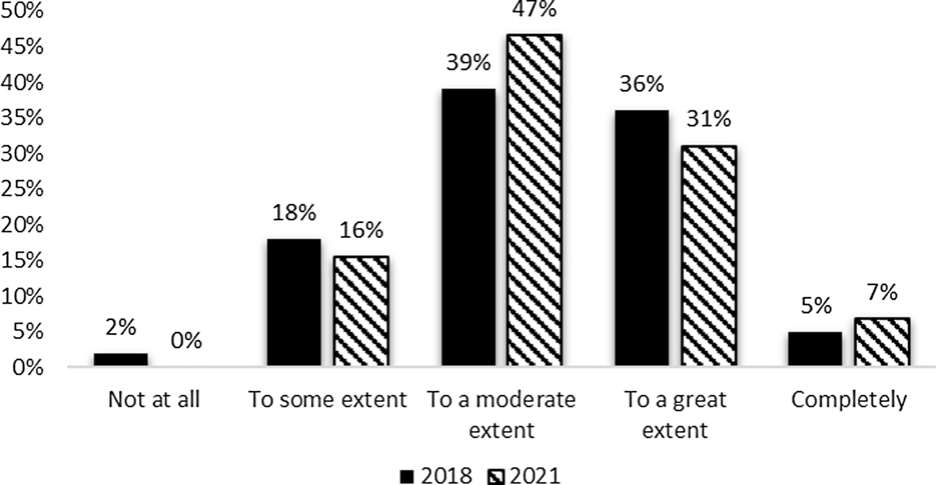
Evaluation contribution of performance improvement decisions 2018 and 2021.

Additionally, the survey requested information to indicate the extent to which the evaluation team was integrated with the leadership of the CTSA hub. Respondents indicated at least some influence on performance improvement decisions. Moderate influence (47%) and great influence (31%) received the most mentions. The pattern of reported influence was similar between the 2018 and 2021 surveys (Fig. 2) with a small increase in reported influence in the most recent survey.

**Figure 3.**
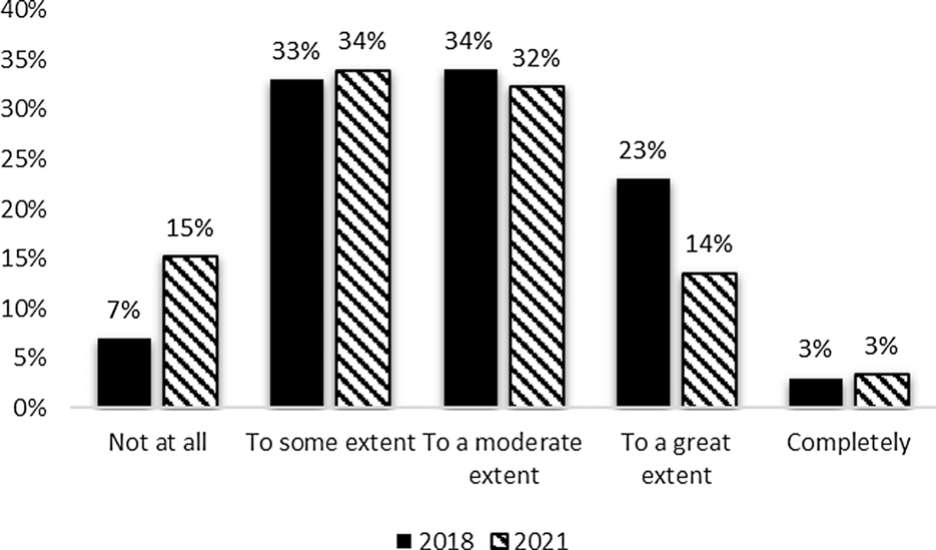
Evaluation contribution to resource allocation decisions 2018 and 2021.

In terms of resource allocation decisions, there was a slight decrease between the 2018 and 2021 surveys in evaluation data’s influence with moderate (32%) and some influence (34%) being most common. However, hubs also occasionally reported that evaluation data had no influence on resource allocation decisions. The responses were similar between the 2018 and 2021 evaluator surveys (Fig. 3) with the exception of the increase in the percent of respondents reporting no contributions from their evaluation data to resource allocation decisions in 2021.

### Evaluation tools and techniques

The third section of the survey focused on evaluation tools and techniques. In addition to availability and use of evaluation expertise, the survey sought information on the use of a selected set of strategic planning or management tools related to evaluating hub performance.

### Evaluation tools

Respondents were asked to report on the use of a list of internal strategic planning tools: logic models, milestones, process models, formal evaluation plans, business process improvement methods, NCATS Common Metrics, and balanced scorecards. They were asked if each tool was in use, in development, or not used. As can be seen in Table 2, in 2021 the NCATS Common Metrics (which was mandatory but has now been sunsetted) was the most frequently reported tool on the list followed closely by formal evaluation plans and the use of milestones. The use of formal business process improvement methods, including balanced scorecards, was reported by less than one-half of the hubs. These results were largely consistent with those found in the 2018 iteration of the survey.

**Table 2.** Hubs’ use of strategic planning tools

Type of Strategic Tool Used	2018 %	2021 %
National Center for Advancing Translational Sciences Common Metrics	NA	98
Formal Evaluation Plans	79	92
Milestones	75	86
Logic Model	62	80
Process Models	36	61
Business Process Improvement Methods	33	37
Balanced Scorecards	NA	36
Other	20	9

NA: this method was not included in the 2018 survey.

### Dissemination techniques

Among the NCATS CTSA Program goals is the advancement of CTS. NCATS has stipulated its expectations that CTSA hubs will develop, demonstrate, and disseminate scientific and operational innovations that improve the efficiency and effectiveness of clinical translation from identification to first-in-human studies to medical practice implementation to community health dissemination. The survey included questions regarding the generation, dissemination, and use of evaluation reports to assess the evaluation teams’ contribution to addressing the dissemination challenge. In terms of outputs, the three most frequently mentioned evaluation outputs were evaluation reports/summaries (93%) and presentations (91%), followed by manuscripts (66%). Flyers and handouts, white papers, and social media posts were all produced by less than one quarter of evaluation teams.

In terms of audiences for evaluation outputs, the three most frequently reported ways to disseminate evaluation products were through meetings with PI/Leadership (97%), Internal Advisory Board meetings (72%), and through email (66%). The use of white paper repositories was least common (5%). Approximately 40% of hubs shared their evaluation output through community-oriented conferences and about 60% used professional conferences as a way of dissemination. Peer-reviewed publications were mentioned by 55% of hubs as a way of dissemination of evaluation outputs.

Consistent with the results described immediately above, the 2021 survey found that evaluation outputs are mostly used to inform hub leadership and specific key stakeholder groups. The most frequently reported users of all evaluation outputs are CTSA Leadership (between 50% and 94%) and CTSA core leadership. This indicates the contribution of the evaluation team in informing hub decision-making. Local researchers were cited as users of flyers/handouts, social media posts, and newsletters by between 50% and 64% of hubs. Local community members were indicated as users of social media posts. newsletters, and flyers by between 50% and 83%.

## Discussion

The CTSA evaluation teams are characterized by being small in size, having high educational attainment, and being a highly collaborative workforce. CTSA evaluators are well-versed in advanced methods, tools, and frameworks but the use of these evaluation skills and tools is uneven within and across CTSA hubs: for instance, Bibliometrics methods are only used by 67% of small hubs whereas 86% of mid-size and 89% of large ones. 67% of small hubs use “machine learning and AI approaches” while only 36% and 44% of mid and large ones do so too. This may well have to do with the shift in priorities at the national leadership level or the greater participation of other hub units in resource allocation and decision-making. In the near future, we expect to see changes in the skills and use of evaluation tools as a result of the new FOA“s focus on continuous quality improvement while at the same time emphasizing an overall push for translational science. As the 2021 survey found, there are still pending issues to be resolved internally but there are other external changes (beyond the CTSA consortium) that must be reckoned with. Given increasing focus on *Open Science* across all federally funded research agencies, CTSA evaluators must improve on their dissemination and communication of products and outputs to the larger community [20]. Better and more effective methods of data collection processes for any data related to diversity, equity, and inclusion (DEI) are also needed (see supplemental material for more). It does not suffice to have a vocal commitment to improving DEI when there is no data (and especially good quality data) to track these issues [21].

It is interesting to note, and this is correlated to the evaluators” profile, that the evaluation work done at the CTSA level continues to remain almost exclusively within the academic world and scholarly production. There is ample room for improvement in going beyond our peers and more into the general public in a more serious effort to truly follow the translational science paradigm from bench to bedside. However, the limited resources currently allocated to evaluation teams within the hubs may well prevent this from happening [22]. Evaluators should consider drafting and committing to external communication plans with support from their hubs administration and communication teams and move beyond the purely internal evaluation realm.

Findings from prior installments of the CTSA Evaluators Survey have already shed light on several initiatives that could be undertaken to strengthen Evaluation across the hubs, namely, a focus on better data to inform decision-making and programing; the importance of providing transparent and utilitarian feedback; an appeal to leverage existing data in more efficient manners; a push to continue building evaluation infrastructure at the local and national level; as well as the potential to leverage and learn from the now-sunsetted “Common Metrics” initiative. Although anecdotal evidence shared through the regularly, and voluntarily convened CTSA evaluator meetings suggests that real efforts have been made in addressing these issues, the reality is that these are still fertile grounds for improvement.

### Recommendations for further enhancing evaluation across the CTSA consortium

#### At the national level

1. There remains a need to provide a national, strategic plan for evaluation. Although there is a mandate for all hubs to provide evaluation services, there are no consortium-wide guidelines that could serve as a unifying theme for evaluation teams. This would aid priority setting and consolidate work towards common goals. The recent creation of the Office of Program Evaluation, Analysis and Reporting seems to be a step in exactly this direction.
2. Central coordination and communications among the 60 evaluator hubs are still required. The logistical problems identified during the deployment of the 2021 survey (i.e. having a current, reliable listing of all evaluation team leadership) must be resolved to facilitate interactions and communications among evaluators and other collaborators.
3. Enhanced Data-sharing, cross-collaboration, and dissemination of evaluation products to increase transparency are needed. The CTSA Evaluators are ideally positioned to lead data-sharing and cross-collaboration initiatives within and across the CTSA Consortium. More deliberate efforts to engage efficiently and productively in making use of existent data, as well as promoting novel data-driven approaches should be encouraged and adequately supported by local hub and national leadership. Dissemination of evaluation outputs to traditional (97% reported dissemination to PI and/or hub leadership) and nontraditional audiences (40% mentioned dissemination to community-oriented groups), as intrinsic to the translational science continuum, must happen more often and consistently across all hubs.

#### At the group-level

1. The CTSA evaluators need to strive to be more inclusive and to continue expanding our collaborations to external nontraditional partners: only 2% of “Other” collaborations were reported; these “Other” included community organizations, state health and Medicaid departments, healthcare organizations, etc. Interdisciplinarity, diversity, and teamwork enhance the quality of the research enterprise. The impact of evaluation can be exponentially increased by adapting this well-established maxim to our own work.
2. Focus on mentoring and training the next generation of evaluators. The 2021 CTSA evaluators survey solicited voluntary participation in mentoring newcomers. Twenty-nine respondents indicated a willingness to serve as mentors. We strongly urge NCATS to contribute resources for turning this voluntary commitment into an officially established program in the near future.

Rigorous, consistent, and, especially, well-coordinated, collaborative cross-hub evaluation processes are necessary to continue advancing the translational science mission of the CTSA program and NCATS. The CTSA evaluators know the importance of common practices, data dissemination, and standard metrics but the participation of this group in leadership and resource allocation discussions is rather heterogeneous. Evaluators bring a breadth of expertise and knowledge that would benefit the entire consortium if brought in consistently to strategic and mission-defining discussions at both the local and the national levels.

CTSA Evaluators take continuous improvement and feedback seriously. The fifth installment of the National Evaluators survey is, once again, proof that as a community, evaluators value consistently and continuously investigating what processes, methods, tools, and best practices are being employed by their colleagues. We believe that periodically reviewing the evaluation capacity of a large infrastructure consortium is a sign of healthy, self-critical engagement with peers and institutional leadership.

## Conclusion

The findings of the 2021 survey show, in many instances, remarkable stability in evaluative capacity, despite external factors such as the COVID-19 pandemic or changes in the FOA, but recognizing larger (i.e. Open Science at the federal level) and even structural changes (i.e. more diverse population) means that evaluators need to continue adapting, improving and responding to all challenges.

## Supporting information

Hoyo et al. supplementary material Hoyo et al. supplementary material

## Supplementary material

The supplementary material for this article can be found at https://doi.org/10.1017/cts.2024.526.
