# Supplementary material for: A landscape assessment of CTSA evaluators and their work in the CTSA consortium, 2021 survey findings
Source: J Clin Transl Sci. 2024 Apr 25;8(1):e79. doi: 10.1017/cts.2024.526 (PMC11091919; doi:10.1017/cts.2024.526)
Supplement: Hoyo et al. supplementary material [file S2059866124005260sup001.docx]

**Supplemental Materials**

Due to space limitations, we wanted to provide here the following additional information about the CTSA evaluators survey.

| Theme | Additional Information |
| --- | --- |
| Survey instrument review and deployment mechanism | The survey underwent review at UCSD’s Human Research Protections Program, and it was deemed exempt from IRB review, continuing review and approval not required under 45 CFR46.104(d), category 2. The final survey instrument was implemented using Qualtrics software, an online survey application (Version July 2021, first release: 2005, Copyright year: 2022, Location: Provo, Utah, USA). |
| Hurdles to completion | The biggest hurdle to completion was a technological challenge. Shortly after the deployment of the survey link via email, the deployment team realized that institutional firewalls and other anti-virus protections at the hub-level were blocking access to the appropriate contact person. Several informational campaigns were undertaken (announcements in group meetings, listservs, etc.), all coordinated through the Program Evaluators Working Group and with the help of CLIC. |
| Year groupings | Year groupings were: 2006, 2007, 2008 and 2009-2010; 2011-2012 and 2013-2019’ the latter three multi-year groupings were such due to the decline in the number of hubs per year and to preserve anonymity of their identities. For the sizes, PAR-15-304 classifications based on direct costs were employed, whereby small means <$4.5 million; medium is between $4.5 and <$6 million and large is >$6 million. |
| Data Analysis | All analyses presented include descriptive statistics only. Given the small sample size, lack of raw data from prior surveys and de-identified datasets, no comparative data were statistically tested. Details about comparative data are available from the authors upon request. |

| Theme | Additional Information |
| --- | --- |
| Examples of CTSA Evaluators collaborations through the lenses of Adaptive Capacity | All the papers cited in the special volume on Adaptive Capacity leveraged existent data- and established data collection processes- to gauge the CTSA hubs’ (individual and collective) ability to adjust and respond to the pandemic. A few specific examples may serve to illustrate the nature of these collaborations, from the local to nation-wide.  Speaking to multi-institutional partnerships was SOCCER (Southern California Consortium of Community Engagement Resources), a joint effort among several CTSA hubs with the goal of bringing underrepresented minorities into research and emergency response, see “Integrating special and underserved populations…” p. 5.  Additionally, and perhaps easier to see given its magnitude and impact is the resulting CTSA-wide collaboration and beyond (NCATS, CD2H and IDeA-CTR) of N3C, described in “Using informatics to advance translational science …”, p.3. |
| DEI item | The 2021 CTSA evaluators survey included a single item on DEI data collection: respondents were asked if they tracked race/ethnicity/gender data for hub participation outside of the mandatory -and education related- tracking. |
| Additional survey items not included in text: Impact questions | Although not included here at length because of limited space, the 2021 survey asked some open-ended and closed questions about Impact.  Despite the fact that 72% of respondents claimed impact evaluation is a priority, only 44% declared having standardized set of impact goals or metrics. This is an example of an issue where coordinated, central guidelines would be useful. What does impact mean for NCATS leadership as it relates to the CTSA mission? |
